# Did internet usage reduce the impact of COVID-19 on the mental health of middle-aged and older adults? A difference-in-differences study based on CFPS data

**DOI:** 10.3389/fpsyg.2024.1462398

**Published:** 2024-10-29

**Authors:** Bo Yang, Xiaofeng Wang, Yuan Zhao

**Affiliations:** School of Public Administration, Northwest University, Xi’an, China

**Keywords:** internet use, mental health, middle-aged and older adults, COVID-19 pandemic, PSM-DID method

## Abstract

**Introduction:**

Mental health is the cornerstone of public health, especially where middle-aged and older adults during the COVID-19 pandemic were concerned. The impact of Internet usage on mental health in the time of the crisis still presents a mixed picture.

**Methods:**

This study employs the PSM-DID method according to longitudinal data (CFPS) to explore whether Internet usage reduced the impact of COVID-19 on the mental health of middle-aged and older adults.

**Results:**

The results reveal that Internet use improves the mental health of middle-aged and older adults during the pandemic in China, but that the impact varies between urban and rural residents. Furthermore, the moderator effects model shows that people’s perceptions of the Internet have an impact upon the length of time spent online and its consequent effect on mental health.

**Discussion:**

These findings suggested that Internet use had a significant effect on alleviating the levels of depression in middle-aged and older adults. Greater marginal gains may be realized by enhancing the digital capacity of and narrowing the digital divide that exists among rural residents. The enhancement of digital capacity and proper guidance in digital education should be taken into consideration where the mental health of middle-aged and older adults is at issue.

## 1 Introduction

The COVID-19 pandemic exerted a statistically significant effect on the mental health of the general population, with the impact ranging from moderate to severe (Daly et al., 2022; Zhang et al., 2023; Brooks et al., 2020; Zhou Y. et al., 2022; Twenge and Joiner, 2020). As for middle-aged and older adults, owing to higher mortality rates from the COVID-19 infection and the typically greater severity of their symptoms (Van Gerwen et al., 2021), they turned out to be most acutely affected and vulnerable group during the pandemic (Tyler et al., 2021; De Pue et al., 2021). When compared with younger adults, older adults exhibited higher rates of fear, distress and depression (Krendl and Perry, 2021; Talevi et al., 2020; Fujita et al., 2021).

Social distancing during the COVID-19 pandemic significantly circumscribed physical activity among middle-aged to older people, especially in those who faced increased risk from chronic disease (Zhou W. et al., 2022). Simultaneously, after having taken into account pre-COVID-19 trends, the experience of loneliness presented a risk factor in worsening mental health during the pandemic (Creese et al., 2021). Face-to-face contact within a social network mitigated the likelihood of experiencing a deleterious state of mental health (Litwin and Levinsky, 2022). Restrictive measures such as quarantining, voluntary isolation, and social distancing impacted upon the psychological wellbeing of people as well as influencing their emotional reactions to the pandemic itself (Talevi et al., 2020). Isolated older people may have developed or experienced an aggravation of mental health conditions because of their isolation (Grolli et al., 2021). Instances of patients reporting concerns for their own physical and mental health declined significantly during this period of “cocooning” (Bailey et al., 2021). What is more, whether older adults lived alone or within a shared household, the perception of stress increased (Ward and Kenny, 2020). The Internet has become an important channel for the public to obtain a variety of health information in China (Xu et al., 2022). The existing literature has revealed the benefits of internet usage among the old, such as decreasing their sense of isolation and depression (Lam et al., 2020), improving their cognitive health (Yuan et al., 2022), and helping them learn about the risk of disease (Li et al., 2022). Meanwhile, the advent of telemedicine has brought new perspectives for health, it can go beyond clinical monitoring and reach aspects related to promoting sports activities (DaCruz et al., 2022). For example, home-based exercises that are suitable for remote implementation can significantly improve the physical and mental health of the older adults (D’Oliveira et al., 2022). Nevertheless, in the era of COVID-19, the transition to telemedicine to cater for mental health care disproportionately impacted the elderly population because the majority of older adults had limited access to or were unable to utilize smartphones and Internet services effectively. Vulnerable older adults have tended to face challenging in making use of digital services owing to lack of Internet connection or their difficulties in mastering online technology. Thus, they have come to fall victim to the double burden of social and digital exclusion (Tsamakis et al., 2021; Seifert et al., 2021).

There is widespread evidence that the COVID-19 pandemic substantially increased Internet use in middle-aged and older adults, which had a significant impact on their lifestyle and mental health (Wu B. et al., 2023; Wang et al., 2022b). According to people’s own self-assessments, Internet use promotes a substantial improvement in self-rated health physical health (Zhou et al., 2023), mental health (Chen and Miao, 2023), subjective wellbeing (Wu Z. et al., 2023; Shi et al., 2023), and quality of life by promoting in turn offline social interaction (Cheng et al., 2023; Wang et al., 2022a), enhancing satisfaction (Cheng et al., 2023) and reducing levels of depression and loneliness (Zhang et al., 2021; Hu and Yu, 2022; Guo and Zhu, 2022). Social media (such as Facebook, WeChat) can in fact help to build trust and happiness (Zhang and Liang, 2023; Ding et al., 2022) and encourage future social participation (Teng, 2022; Du et al., 2023).

One cluster of research has identified that the increased use of the Internet during the pandemic had a negative impact on the mental health of middle-aged and older adults. The Internet remains a crucial channel for accessing health-related information (Du et al., 2020). It has been shown that the COVID-19 period witnessed a spike in Internet searches relating to topics of fear, anxiety, and depression, as well as seeing users seek out knowledge about health in general. Internet use affects the mental health of older adults and increases incidences of depressive symptoms (Xie et al., 2021). The prolonged use of social networking sites (SNS), watching short videos and shopping online may nurture loneliness and the symptoms of depression (Pantic, 2014; Zheng et al., 2023). Correlation analysis has revealed that Smartphone addiction is positively correlated with depression and anxiety on a palpable level (Karaş et al., 2023). The WHO has advised the public to focus less on news relating to COVID-19 and to spend minimal time online as a means of alleviating the effects upon mental health (WHO, 2020). This is especially timely because the public often lacks the appropriate level of knowledge to be able to distinguish fake information from genuine scientific updates for most of such information about illness is often derived from preliminary observations, and hence is often unreliable and speculative. However, it leads to lots of confusion and anxiety due to lack of basic disease prevention knowledge and proper health-related information from social media and private unfiltered networks like Facebook, Twitter, YouTube, WeChat, TikTok, etc. (Patel et al., 2020).

The growing utilization of the Internet may encourage citizens, especially those who have a higher digital capacity, to spend increasing time online. Worry has thereby grown about its effects upon the mental health of and depression among older adults (Nakagomi et al., 2022; Fu et al., 2023). Previous studies have proved that there is a U curve-shaped relationship between the duration and frequency of Internet use and levels of depression (Zhang et al., 2022; Hu and Yu, 2022; Zhang et al., 2022). However, the precise underlying mechanism of this U curve-shaped relationship is still unclear. The duality evinced by the Internet underscores the need for a balanced approach to web usage, whereby informational benefits are maximized while potential mental health risks are mitigated.

Based on cognitive emotion theory and emotion-action link (Verhagen and van Dolen, 2011; Oatley & Johnson-laird, 1987), this paper tests the role of the perception importance of Internet plays in emotion and Internet use. Cognitions are essential in shaping the essence of emotions (Folkman and Lazarus, 1985). Emotions arise from the dynamic interaction between an individual and their environment, whereby personal assessments of perceptions catalyze behavioral and physiological shifts. This process underscores the pivotal role of cognitive appraisal in the emotional response system, highlighting how individuals’ interpretations of experiences fundamentally influence the emotional landscape. People’s perceived assessment of the importance of the Internet has a significant impact on the role it plays in their lives.

In line with cognitive emotion and emotion-action link theory, the online environment can offer users a highly stimulating and rewarding experience that enhances positive emotion and increases the amount of time they wish to spend on the Internet. The content found online, including music and videos, social information, and games, together with Internet activities such as online social networking, social interactions with family, friends, and others, not to mention shopping are inherently pleasurable in nature (Greenfield, 2011; Young, 1998). These can serve to alleviate depression (Gaia et al., 2021; Jin and Zhao, 2019; Neves et al., 2018). The Internet paves the way to accessing content and enjoying frequent and convenient engagement. Users receive pleasurable feedback in the form of Facebook or WeChat “likes” or blog views and comments. The propensity of the Internet to generate such feedback encourages excessive online usage and can give rise to problems and abusive behavior (Kuss et al., 2014; Brand et al., 2014).

The internet has played an essential role in the mental health of older adults, especially during the COVID-19 epidemic. Owing to the dual nature of Internet use, studies exploring its effect upon mental health have yielded mixed results. On the one hand, existing studies have shown that the Internet has had a positive impact on mental health. On the other hand, contrasting studies have provided evidence that the Internet to some extent weakens mental health.

Based on the previous studies, the present study aims to explore if Internet usage could alleviate the impact of COVID-19 on the mental health of middle-aged and older adults. Firstly, using full sample and subsample (Internet users and nonusers), we set a basic regression model to explore the impact of the COVID-19 pandemic on the mental health of middle-aged and older adults. Secondly, the endogeneity problems caused by the issue of self-selection bias and omitted variables in the OLS model are addressed by using the difference-in-differences (DID) method. The DID method is used to explore whether Internet use could alleviate the impact on depression level. Furthermore, the study examines how particular patterns of internet usage may influence the levels of depression among middle-aged and older adults. Thirdly, in the heterogeneity analysis, we compared individuals from rural and urban areas in order to provide further analysis of the rural–urban divide on the impact of Internet usage on the mental health of middle-aged and older adults during the COVID-19 pandemic. Lastly, utilizing cognitive emotion theory and emotion-action link as theoretical frameworks, the study examines whether the perception of Internet acts as a mediating variable in the time spent online and thus on the mental health of this group.

In due course, several incidental contributions are made to existing research. First of all, existing research generally uses cross-sectional data and employs PSM, OLS, and the ordinal OLS method, which may lead to estimate biases and overlooked variables. Fewer studies have employed the DID method to explore the effects of Internet on health. In order to deal with the endogenous issue of Internet use and mental health, the PSM-DID method is employed in this paper. Secondly, various studies have reported physical exercise, social participation, and life satisfaction as playing positive roles in the mental health of the elderly. They are considered as a stimulus which increases the level of cognitive function and decreases the sense of loneliness felt during the COVID-19 pandemic (Xin and Ma, 2023; DaCruz et al., 2022; Zhang et al., 2022). Even so, less research has focused on the perception of the Internet as being a mediator bridging the Internet use and the mental health. Based on the theory of cognitive emotion and emotion-action link, this paper investigates the mechanism underlying the U curve-shaped relationship plotting time spent online and the mental health of the middle-aged and older adults. Thirdly, the relationship between the Internet and the mental health of older adults has become a hot topic. Even so, the casual effect of this relationship remains unclear. In contrast to previous research, we have selected Internet users as the targeted group, drawing data from the 2018 and 2020 rounds of the China Family Panel Studies (CFPS). This study performed difference-in-differences analysis to explore the casual impact of internet usage on mental health of older adults, especially with respect to whether Internet usage alleviated depression among older Chinese adults during the COVID-19 pandemic, which furthered the cross-sectional data research (Zhang et al., 2021; Zheng et al., 2023; Zhu et al., 2021; Jing et al., 2023). Filling the gap of cognitional perspective of relationship between Internet use and the mental health, this study applied the theory of cognitive emotion theory and emotion-action link to explore the mechanism of it and sought to confirm the applicability of existing theory to the Chinese sample.

## 2 Data and variables

### 2.1 Data

The longitudinal data of this study was obtained from the 2018 and 2020 rounds of the China Family Panel Studies (CFPS). CFPS is a nationally representative, biannual longitudinal survey of Chinese communities, families, and individuals launched by the Institute of Social Science Survey (ISSS) of Peking University, China. Its baseline survey was officially implemented in 2010, involving 25 provinces/cities/autonomous regions across the country, and ultimately completed visits to 14,960 households and 42,590 individuals from 25 provinces/municipalities in China mainland (excluding Hong Kong, Macao, Taiwan, Tibet, Inner Mongolia, Qinhai, Xinjiang, Ningxia and Hainan), representing around 95 percent of the Chinese population. All family members defined by the baseline survey and their future biological/adopted children have been permanently tracked and investigated. Five follow-up surveys were conducted in 2012,2014,2016,2018 and 2020. The CFPS dataset includes a set of questions derived from the most widely used self-report scale, the CES-D scale short version as measured depressive symptomatology in the general population (Radloff, 1977). Thanks to the information, we were able to test how Internet interacts with the pandemic to affect mental health. Our study focused on the effect of COVID-19 on mental health of middle-aged and older adults, the 2018 and 2020 surveys were used for comparation. In the two surveys, participants ranged in age from 9 to 104. For our analysis, we have excluded individuals under the age of 45 and those with incomplete data on key variables. As a result, 27,697 observations were used for analysis after sample selection.

### 2.2 Variables

#### 2.2.1 Dependent variable

Depression was used as the measure for guaging the mental health of middle-aged and older adults as it is a common manifestation and can be expected to be a predictor of mental health. Depression levels in middle-aged and older adults were used to measure for the effect of Internet usage on mental health by adopting the CES-D scale short version of the CFPS questionnaire which included 8 questions (namely: “I feel in low spirits,” “I feel that doing anything is a struggle,” “I’m not sleeping well,” “I feel lonely,” “I feel sad and upset,” “I feel life is unbearable,” “I feel happy,” and “I live a happy life”) with 4 response options (namely: 0 representing never, 1 representing occasionally, 2 representing often, and 3 representing most of the time). Respondents’ depression scores were calculated by adding together the scores from each question and the scores of the last two questions were reverse coded. Higher scores indicated higher levels of depression.

#### 2.2.2 Core independent variable

The China Family Panel Studies (CFPS) questionnaires from both the 2018 and 2020 editions included the following question: “Do you use mobile devices such as cell phones to surf the Internet?” and “Do you use a computer to surf the Internet?” Respondents answered “yes” or “no,” and those who used a computer or a cell phone to surf the Internet were defined as being Internet users and assigned a value of 1. Those who did not use the Internet were assigned a value of 0. Then participants reported four different online activities (“Do you use the internet for the following purposes in past week?”): (1) Entertainment (e.g., short-form video, playing online games); (2) Online shopping; (3) Online learning;(4) social networking (e.g., e-mail, WeChat) and we set the answer to “yes” =1 and “no = 0.”

#### 2.2.3 Control variables

The age, gender, education, and health status of older adults are endogenous to a considerable degree, affecting both their mental health and their Internet use. Hence, they were used as control variables. The variables were defined as follows: the gender of the respondent was taken as 1 for males and 0 for females. Age was defined as the numerical difference between the year the respondent was born and the year when the respondent was interviewed. Education referred to the highest educational level achieved by the respondent, covering three levels (a lower level of education such as illiterate, elementary, junior high school; an intermediate level of education such as high school, technical school, vocational high school; and a higher level of education such as college, bachelor’s degree, master’s degree, doctorate. Sequentially, the values of the three levels were taken as 1–3 points). Health status referred to the respondents’ perception of their health, which was divided into 5 levels, with the lowest value being 1, signifying “unhealthy,” and the highest value being 5, signifying “very healthy.”

#### 2.2.4 Mediator variable

In order to explore perceptions of the Internet, we used the question found in both the 2018 and 2020 questionnaires - “How important is the Internet to your accessing information?” - as a means of assessing their attitudes toward the Internet. This was divided into 5 levels, with the lowest value being 1, signifying “extremely unimportant,” and the highest value being 5, signifying “extremely important.”

## 3 Methods

In order to explore the comprehensive impact of Internet use on the mental health of middle-aged and older adults during the COVID-19 pandemic, we made use of the Panel regression models and the DID models. The outbreak of an epidemic at a certain time can be thought of as a random occurrence or extraneous to other factors determining health. Therefore, we can treat the outbreak as a case study and compare the change in health among middle-aged and older adults so as to investigate the causal relationship between Internet use and mental health.

In this paper, the endogeneity problem caused by the issue of self-selection bias and omitted variables is addressed by using the difference-in-differences (DID) method. This method was first proposed by Heckman et al. (1997) for assessing the effects of public policy. As a quasi-experimental analysis method, the approach is frequently employed to track the changes resulting from a new event or policy effect over time. It provides a systematic means to observe and measure the outcomes that arise from shifts in policy or circumstances, offering valuable insights into their long-term efficacy and implications. The difference between the core observed variables before and after the pandemic is calculated by dividing different individuals who are the internet users, and those who are not, into a treatment group and a control group. The pure effect is obtained by eliminating the fixed effect and the influence of common time trends among middle-aged and older adults. In contrast to other experimental designs, the DID method effectively addresses the issue of omitted variables that remain constant over time. It also mitigates the influence of external factors and biases stemming from the effects of selection, providing a more robust analysis of the causal impact of policy changes or interventions. Given these advantages, the DID method has become a staple for the quantitative assessment of the impacts stemming from the execution of specific public policies or initiatives. It enjoys widespread application across the discipline of econometrics and has also gained significant traction in the field of sociology.

Two assumptions are brought into play when it comes to the DID method (Dehejia, 2005): the common trend and the randomized assignment of group status. The common trend requires that both the Internet users group and the non-users group have exhibited the same trend of change over time, and that this can be verified using the common support test. The use of the Internet is sometimes a matter of self-selection, influenced by factors such as educational background and socioeconomic status. This makes it inappropriate to consider non-users as a counterfactual sample for Internet users, as the selection process is not random. As a means of solving these problems, this study employs the propensity score matching method (PSM) (Heckman et al., 1997) for supplemental improvements to the DID method.

## 4 Model

In the first part, we performed panel regression as baseline model to estimate the effect of COVID-19 pandemic different specifications of the linear econometric model:


(1) $$Y_{ik}=\alpha +\beta \mathrm{COVID}\;+\;\eta \;X_{ik}+\varepsilon_{ik}$$

In Equation 1, $Y_{ik}$ is the depression score of individual I at k year using the CES-D as defined above in 2018 and 2020 waves; COVID is a dummy where equals to 1 if the responder has experienced epidemic in 2020 or is interviewed in 2018. $X_{ik}$ is a vector of demographic variables, including age, gender, education, self-rated health and socioeconomic status. Finally, $\varepsilon_{ik}$ is the error term of the regression.

In the second part, The DID model was set to solve the endogeneity problem mentioned above:


(2) $$Y_{ik}=\beta_{0}+\beta_{1}dU_{ik}+\beta_{2}dt_{ik}+\beta_{3}dU_{ik}*dt_{ik}+\varepsilon_{ik}$$

In Equation 2, $Y_{ik}$ is the explanatory variable, representing the internet effect on the mental health of individual I at year k. $\beta_{1}$ indicates the gap in the middle-aged and older adults’ mental health itself; Treatment indicates whether or not to use the Internet, and takes the value of 1 for using the Internet and 0 for not using the Internet; $\beta_{2}$ indicates the time effect; time indicates the period when middle-aged and older adults are using the Internet, and takes the value of 0 to denote before the Internet was used and 1 for after the Internet was used; $\beta_{3}$ represents the pure effect of change in mental health after removing the time effect and the disparity in individuals themselves; and $\varepsilon_{ik}$ indicates the error term.

In previous studies, the impact of Internet use on the health of older adults was often generalized by treating this demographic as a homogeneous group. However, it is important to recognize that there is a significant degree of heterogeneity in Internet usage among older adults, which can be attributed to their diverse individual habits and characteristics. In the study, demographic variables such as gender and age, Internet use, levels of depression, and the life satisfaction of elderly individuals were first described in order to obtain a basic picture of the data. Secondly, the DID method was used to determine the causal relationship between the use of the Internet and changes in the mental health of middle-aged and older adults from 2018 to 2020. Furthermore, the pure effect of Internet use on the mental health of elderly individuals was evaluated based on the difference in the change.

When conducting a PSM analysis, we used pre-treatment data to estimate the probability that each observational unit would receive this treatment. This probability is referred to as the propensity score. Specifically, Logit or Probit regression models can be used to estimate the propensity score. In this step, the treatment variable (i.e., whether or not to use the Internet) is used as the dependent variable, and the covariates (other variables that may affect the allocation of treatment) are used as independent variables. The model typically takes the form:


(3) $$\mathrm{Logit}\left(dU_{ik}=\eta \right)=\beta_{0}+\beta_{1}X_{i1}+\beta_{2}X_{i2}+\cdots +\beta_{k}X_{ik}$$

In Equation 3, $dU_{ik}$is the dummy variable of Internet use. When *η* = 1, it means that this individual is an Internet user; when η = 0 represents that the individual is a non-Internet user. The covariates $X_{i1},X_{i2},\cdots X_{ik}$ encompass age, gender, education, and health status. Individuals in the same value range were matched after using kernel matching methods.

## 5 Results

### 5.1 Descriptive statistics

Previous research has showed that constant and new Internet use had different effects on depressive symptoms (Jing et al., 2023). To compare the impact of the Internet use on mental health, Internet users can be categorized into distinct groups: Group A comprises adults who had internet access in both 2018 and 2020, while Group B consists of adults who were non-Internet users in 2018 but began using the internet in 2020. Group C comprises adults who were non-Internet users in both 2018 and 2020, thereby forming the control group. Table 1 shows the descriptive analysis of Internet users and non-users. Both in 2018 and 2020, the age profile of Group C was older than that of Group A and Group B, with males having greater access to the Internet than females. Also, the average educational level of Group A and Group B was higher than that of Group C. It is worth considering that the depression score for middle-aged and older adults in 2018 was higher than that of samples in 2020. The depression levels of middle-aged and older adults were influenced by variable factors and the self-selection of samples may have led to biased results. As a result, the current study adopts panel data, sets up PSM-DID model to overcome the problem of endogeneity.

**Table 1:** The descriptive analysis on variables.

	2018			2020		
	Group A	Group B	Group C	Group A	Group B	Group C
Variables	N%, Mean (S.D)	N%	N%	N%	N%	N%
		Mean (S.D)	Mean (S.D)	Mean (S.D)	Mean (S.D)	Mean (S.D)
Age	53.43	54.81	63.55	55.43	56.8	63.54
	(7.24)	(7.43)	(10.24)	(7.24)	(7.41)	(10.24)
Gender						
Female	44.79	47.8	53.8	44.71	47.88	53.77
Male	55.21	52.2	46.2	55.29	52.12	46.23
Education						
Low	23.47	44.4	72.12	25.58	47.31	75.78
Middle	62.69	52.68	27.02	61.07	50.07	23.49
High	13.84	2.92	0.86	13.35	2.62	0.73
Self-rated health						
Unhealthy	16.21	20.41	30.5	16.32	19.46	30.06
Generally healthy	14.15	15.49	16.85	12.03	12.05	16.12
Relatively health	50.57	40.83	33	49.48	43.05	32.56
Very healthy	9.99	11.49	9.42	11.72	11.62	10.29
Extremely healthy	9.08	11.78	10.23	10.44	13.83	10.98
SES						
Low	8.45	7.79	7.54	7.42	5.97	6.26
Lower-middle	14.48	12.18	11.53	14.05	12.58	10.65
Middle	48	44.4	37.67	49.82	44.56	36.65
Upper-middle	21.06	20.95	20.56	20.68	22.25	22.58
Upper	8.01	14.67	22.69	8.02	14.64	23.86
Depression scores	13.07	13.53	14	13.07	13.32	14.09
	(3.77)	(4.04)	(4.47)	(4.09)	(4.26)	(4.57)

### 5.2 Test of model assumptions

Before we proceeded to use the PSM-DID methodology to assess the average treatment effect brought about by Internet usage, we tested whether Internet usage has contributed significantly to the health of middle-aged and older adults during the COVID-19 pandemic. It is essential to obtain propensity score matching values from which Internet users and non-users are kernel-matched; testing the quality of the match in the previous step, i.e., whether the common trend and random grouping assumptions were met (Heckman et al., 1997; Rosenbaum and Rubin, 1983). This includes a balance test and a common support test to examine whether the matched samples of the experimental and control groups overcome the problem of self-selection and whether there is a significant difference between the means of the two covariates.

We report the main results of this paper in three parts. In the first part, we report baseline results, which test the effects of Internet on the mental health of middle-aged and older adults. The second part reports the results of mental health of Internet users and non-users’ heterogeneity analysis, which addresses the difference between rural and urban residents. The third part investigates the efficacy of the uncertainty mechanism.

#### 5.2.1 The quality of matching

The propensity scores matching (PSM) estimation results are shown in Table 2. Most of the *p*-values of covariate coefficients were significant (*p* < 1%), indicating that they are key factors. The *p*-value in the model is greater than chi2 value in the Wald chi-square test, therefore, the model is significant and the propensity score matching was proved to be successful.

**Table 2:** The quality of matching.

Covariate	Coefficient	z	p > |z|
Education	1.845	45.62	0.000***
Age	−0.118	−40.25	0.000***
Gender	0.009	0.2	0.083*
Self-rated health	0.039	2.16	0.031**
SES	−0.170	−8.49	0.000***
LR chi2	5753.67	Prob>chi2	0.000***

#### 5.2.2 The balance test of the PSM

**Figure 1:**
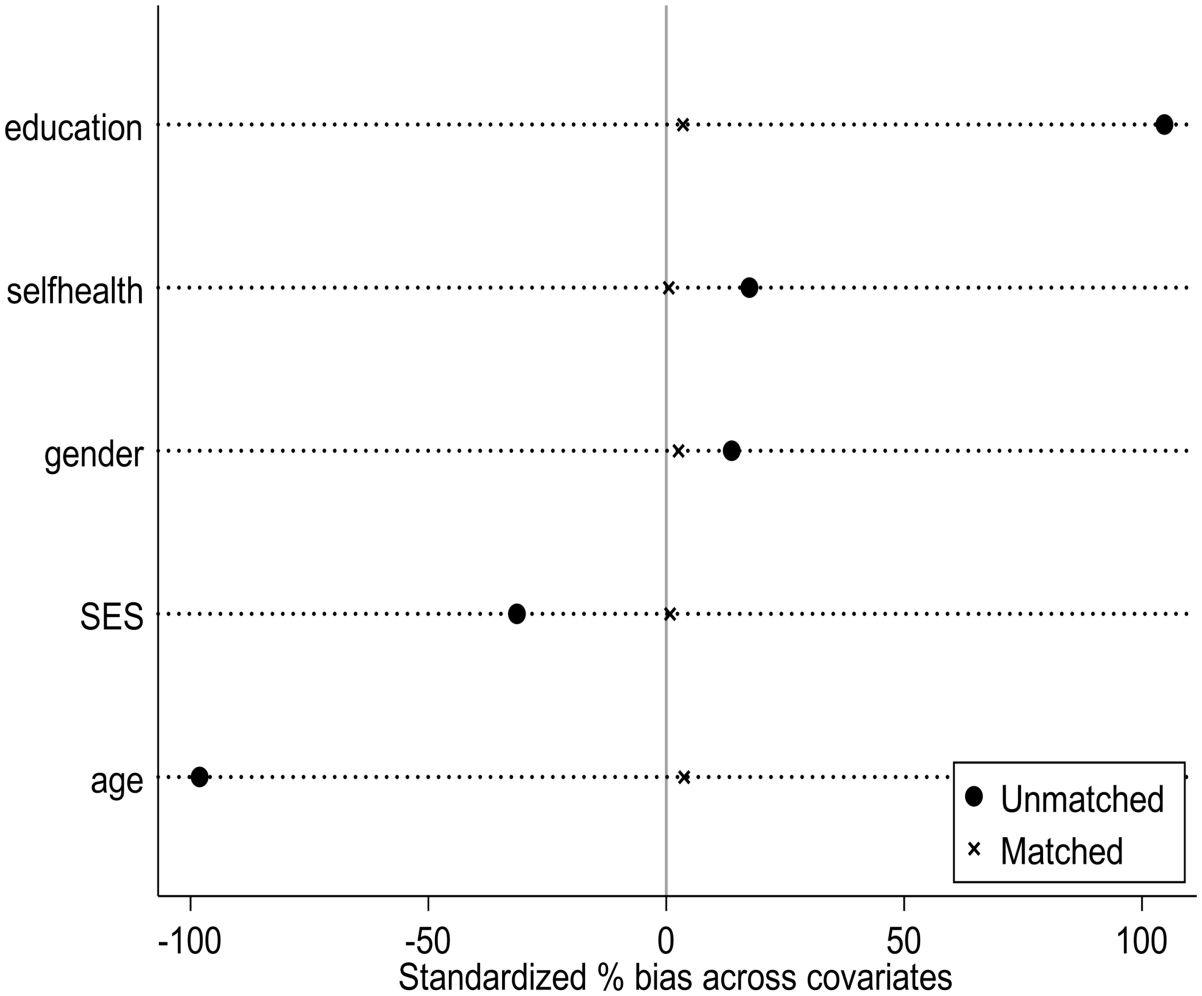
Standardized % bias across covariables.

Following the sample matching process, a balance test was conducted so as to verify that the data was a well-balanced and approximate random selection, to be used in the subsequent regression. This entailed ensuring that there were no significant differences in the covariates and propensity scores between the treatment and control groups, thus eliminating discernible discrepancies that could bias the results. The results of the sample matching quality balance test are shown in Table 3. A bias score was calculated for outcome variables. The bias of the standard deviation has dropped to no more than 4%, which is significant and meets the requirement of not exceeding 20%. There was no systematic difference between the treated group and the control group (*p* < 1%). Therefore, the matched samples met the random grouping requirement and matching was completed successfully (Figure 1).

**Table 3:** Sample matching quality balance test.

Variable	Unmatched Matched	Mean		Bias (%)	Reduce bias (%)	*T*-test	
		Treated	Control			t	p > |t|
Education	U	1.865	1.305	104.7	96.7	63.46	0.000
	M	1.865	1.846	3.5		1.62	0.106
Age	U	54.136	62.14	−98.1	96.2	−55.63	0.000
	M	54.136	53.833	3.7		2.15	0.032
Gender	U	0.547	0.478	13.7	81.6	8.03	0.000
	M	0.547	0.534	2.5		1.28	0.202
Self-rated health	U	2.858	2.646	17.5	97.2	9.93	0.000
	M	2.858	2.852	0.5		0.27	0.790
SES	U	3.075	3.412	−31.4	97.6	−17.86	0.000
	M	3.075	3.067	0.8		0.42	0.677

#### 5.2.3 The common support test

Before employing the PSM-DID approach to estimate the effects of an exogenous shock, it is essential to verify if the data meets the common trend assumption. Additionally, it is crucial to assess the extent to which the observations from both the control and treatment groups overlap.

Out of the 15,398 total matched observations, 211 observations in the control group were not in common support, 89 observations in the treatment group did not overlap, and the remaining observed values fell into the common support region. While among the observations in support, 10,333 observations in control group lie in the range of the common trend, and 4,765 observations in the treated group fell in the range (Table 4). The results of the common support test across all variables have proved uniformly affirmative. Consequently, the extensive common support region further substantiates the suitability of the PSM-DID method for this study.

**Table 4:** Results of common support test.

	Off support	On support	Total observations
Control group	211	10,333	10,544
Treated group	89	4,765	4,854
Total observations	300	15,098	15,398

### 5.3 The baseline results

The basic panel regressions were performed to estimate how mental health was associated with COVID-19 and examine potential differences of mental health between those with and without Internet access among respondents aged 45 years and older who completed the questionnaire at 2018 and 2020. Table 5 showed the coefficient of COVID-19 of model 1 is statistically significant at a 1% significance level for all of the middle-aged and older adults. Demographic variables constant, Internet users have depression scores of 0.258 (Group A) and 0.058 (Group B) respectively, whereas that the depression scores for nonusers is 0.217. However, due to the endogeneity problem, we could not explore whether the Internet users are less or more likely to be in a depressed state than are nonusers before and after the COVID-19 pandemic.

**Table 5:** Results of the effects of COVID-19 pandemic on mental health.

Variables	Model1	Model2	Model3	Model4
	Depression level	Depression level	Depression level	Depression level
	Full sample	Group A	Group B	Group C
COVID-19 (No)	(Ref)	(Ref)	(Ref)	(Ref)
(Yes)	0.176*** (0.045)	0.258*** (0.073)	0.058* (0.123)	0.217*** (0.060)
Education	−0.811*** (0.067)	−0.670*** (0.105)	−0.416** (0.187)	−0.750*** (0.083)
Gender	−0.887*** (0.060)	−0.636*** (0.094)	−0.738*** (0.193)	−0.968*** (0.074)
Self-rated health	−0.968*** (0.022)	−0.943*** (0.038)	−0.834*** (0.068)	−1.008*** (0.027)
Age	−0.011*** (0.003)	−0.040*** (0.007)	−0.034** (0.014)	−0.011*** (0.004)
SES	−0.306*** (0.024)	−0.359*** (0.042)	−0.359*** (0.077)	−0.301*** (0.029)
Constant	19.99*** (0.223)	20.86*** (0.389)	20.92*** (0.791)	20.17*** (0.275)

***p < 0.01, **p < 0.05, *p < 0.1.

In order to explore the impact of Internet use on the health of middle-aged and older adults before and after the COVID-19 pandemic, the PSM-DID models were constructed implementing controls based upon age, gender, education, health status and socioeconomic status.

In the light of the tests in the Methodology section, the matched samples met the requirements of the PSM-DID methodology. Consequently, the study used mental health as an outcome variable and obtained the average treatment effect of the Internet use by comparing Group A and Group C, and adding control variables to the model so as to further verify the results. Model 5 in Table 6 represents the results of the regression of the Internet effect and Model 6 the results of regression with control variables introduced. Internet use has been found to significantly influence the level of depression among middle-aged and older adults when compared with the group that did not use the Internet. The coefficient of the PSM-DID model was −0.218, with a significant value of 10%, which indicates that Internet use had a negative effect on the depression levels of the sample population. Middle-aged and older adults who used the Internet reduced their levels of depression by 0.218 points, compared with those who did not use the Internet. Similarly, Internet use had a negatively significant impact on the frequence of middle-aged and older adults.

**Table 6:** Results of the DID estimation.

Variables	Model5 [Group A vs. Group C (ref.)]	Model6 [Group B vs. Group C (ref.)]
	Depression level	Depression level
DID matching estimator	−0.218* (0.130)	−0.426** (0.166)
Education	2.072 (4.126)	1.079 (4.277)
Gender	10.92*** (4.033)	4.611* (2.470)
Self-rated health	−0.511*** (0.062)	−0.478*** (0.067)
Age	−0.098 (0.290)	0.059 (0.146)
SES	−0.188*** (0.064)	−0.129* (0.069)

***p < 0.01, **p < 0.05, *p < 0.1.

In terms of Group B and Group C, the results of the regression of the Internet effect on mental health reveal lower levels of depression among middle-aged and older adults who were new to using the Internet than in the group that did not use the Internet at all. Furthermore, compared with Group A and Group C, Group B showed that Internet usage had a greater influence on depression than did Group C since the coefficient of the PSM-DID estimator was −0.426, with a significant value of 5% while the same estimator of Group A was −0.218 (Table 6).

Furthermore, the effect of different types of online activities on depressive status needs to be considered (Table 7). The results showed that online entertainment (*β* = −0.23, 10% significance) and social networking (*β* = −0.358, 1% significance) were associated with lower depression scores while online shopping and online learning had no significant effect.

**Table 7:** Effects of types of online activities on mental health.

Variables	Depression level			
Online entertainment	−0.23* (0.14)			
Online shopping		−0.215 (0.208)		
Online learning			−0.569 (0.305)	
Social networking				−0.358*** (0.097)
Controlled variables	Yes	Yes	Yes	Yes

***p < 0.01, **p < 0.05, *p < 0.1.

### 5.4 Heterogeneity analysis

Different middle-aged and older adults have their unique characteristics and modes of accessing information. The current socio-economic landscape of China sports a pronounced duality, characterized by significant disparities between its rural and urban sectors. In China, a notable prefectural digital divide is observed, marked by a progressive decline in the ICT development index from the eastern regions to the western areas and from central cities to more peripheral ones. Despite an overall increase in Internet users in both urban and rural China, a considerable disparity remains in the rates of Internet penetration between these two areas. According to Digital China Development Report (2021), China had constructed 1.425 million 5G base stations by the end of 2021, these being concentrated in urban areas. In contrast, the Internet penetration rate in rural areas remains below 60%, having only to date achieved 4G coverage.

Furthermore, there exists a notable disparity in the technological proficiency of Internet users between urban and rural populations. This has raised concerns regarding the potential health implications of digital inequality between these regions, particularly for middle-aged and older adults. This paper compares individuals from rural and urban areas in order to provide further analysis of the rural–urban divide on the impact of Internet usage on the mental health of middle-aged and older adults during the COVID-19 pandemic. Based on the regression analysis presented in Table 8, it is generally observed that individuals in the Internet usage access group and the new entrant Internet group from rural areas exhibit lower depression scores compared to their counterparts of rural Internet nonusers. Conversely, within urban areas, no significant difference in depression scores is noted between individuals who use the Internet and those who do not. This suggests that middle-aged and older adults in rural areas enjoy a greater marginal benefit from Internet usage compared to their urban counterparts.

**Table 8:** The results of heterogeneity analysis.

	Group A vs. Group C (Ref.)		Group B vs. Group C (Ref.)	
Variables	Depression level		Depression level	
	Rural	Urban	Rural	Urban
DID matching estimator	−0.290* (0.171)	−0.404 (0.259)	−0.572*** (0.199)	−0.240 (0.325)
education	2.265 (4.358)	−0.249 (0.167)	1.169 (4.362)	−0.317 (0.210)
gender	10.78** (4.250)	−0.870*** (0.162)	2.924 (3.090)	−1.252*** (0.227)
Self-rated health	−0.521*** (0.070)	−0.862*** (0.071)	−0.462*** (0.076)	−0.837*** (0.092)
age	−0.136 (0.320)	−0.050*** (0.010)	0.345 (0.216)	−0.053*** (0.014)
SES	−0.105 (0.072)	−0.569*** (0.076)	−0.111 (0.081)	−0.431*** (0.098)
R-squared	0.027	0.16	0.024	0.17

***p < 0.01, **p < 0.05, *p < 0.1.

### 5.5 Further analysis

**Figure 2:**
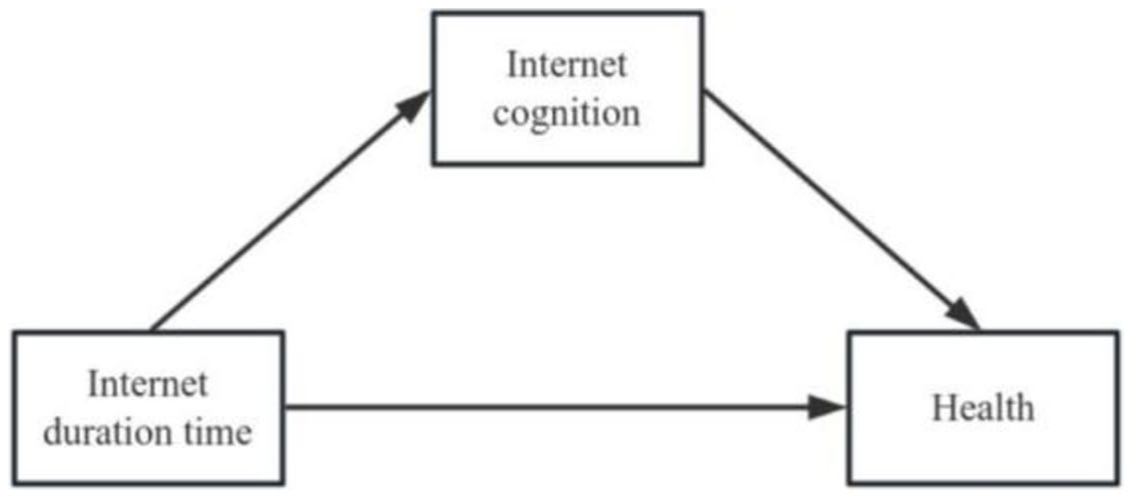
Internet cognition as mediating factor.

The negative relationship witnessed between Internet usage and depression in middle-aged and older adults echoes that discovered in previous research. Even so, the mechanism of impact of Internet usage on the mental health of middle-aged and older adults still remained unclear. To explore the casual relationship between Internet usage and mental health of middle-aged and older adults, we added time spent online and its quadratic term in the regression Equation 4 and Equation 5 is expanded to incorporate the mediation effect test in order to identify the uncertainty mechanism. Based on the theory of cognitive emotion theory and emotion-action link, those who believe that the Internet is an important source of information are liable to spend more time online. The perception of the Internet can serve as a mediator between the length of time spent online and the health of middle-aged and older adults (Figure 2). This study aims to empirically examine whether the perception of Internet acts as a mediating variable in the time spent online and thus on the health of this group.

Considering that the fixed-effects model can effectively mitigate endogeneity issues arising from omitted variables, we constructed the following bidirectional fixed-effects regression model, including Internet usage duration and its square, to provide evidence for a U curve-shaped relationship:


(4) $$\mathrm{Healt}h_{\mathrm{it}}=\beta_{0}+\beta_{1}X_{\mathrm{it}}+\beta_{2}X_{\mathrm{it}}^{2}+\beta_{3}\eta_{i}+u_{i}+v_{t}+\varepsilon_{\mathrm{it}}$$

In Equation 4, *i* represents the individual, *t* represents the year, and mental health, denoted by the dependent variable $\mathrm{Healt}h_{\mathrm{it}}$. The independent variable $X_{i}$, represents the length of time spent online. A set of control variables, denoted by $\eta_{i}$ are included in the model to account for additional influences. $u_{i}$ represents individual fixed effects and $v_{t}$ is the time-fixed effect. The random disturbance term, which captures unobserved heterogeneity, is represented by $\varepsilon_{t}$.


(5) $$\begin{array}{c} \mathrm{Healt}h_{\mathrm{it}}=\beta_{0}+\beta_{1}X_{\mathrm{it}}+\beta_{2}X_{\mathrm{it}}^{2}+\beta_{3}X_{\mathrm{it}}Z_{\mathrm{it}}\\ +\beta_{4}X_{\mathrm{it}}^{2}Z_{\mathrm{it}}+\beta_{5}Z_{\mathrm{it}}+\beta_{6}\eta_{i}+u_{i}+v_{t}+\varepsilon_{\mathrm{it}} \end{array}$$

In order to explore the mediation effect, this paper further constructed a nonlinear mediation effect model to examine the channels through which the Internet affects the health of middle-aged and older adults. In Equation 5, the mediating variable reflecting Internet cognition denoted by $Z_{\mathrm{it}}$. Other parameters remain consistent with those in Equation 4.

From the results reported in Table 9, the coefficient of time spent online is negative and significant after including the quadratic term of time spent online, while the coefficient of the latter term is positive and significant. This indicates that there is a U curve-shaped relationship between time spent online and depression, which echoes the previous research. To test the moderator effects the latent mechanism and in turn, the observed U curve-shaped relationship, interactions between the moderator, attitudes toward the Internet, and time spent online and its square are introduced (Aiken et al., 1991; Haans et al., 2016). The results indicate that a steeping change occurs for the U curve-shaped relationship between time spent online and the mental health of middle-aged and older adults when the coefficient of interaction of attitudes toward the Internet and time spent online is positive. When the time spent online lies in the low range, the attitude toward the Internet enhances the negative effect of the Internet on the depression while after a turning point it enhances the positive impact of the Internet on levels of depression. As a result, it is essential to steer middle-aged and older adults toward healthy Internet usage and online behavior.

**Table 9:** Results of relationship between Internet use duration and mental health.

Variables	Depression level	Depression level
Internet duration	−0.108** (0.555)	−0.380*** (0.146)
Internet duration square	0.026** (0.104)	0.067*** (0.018)
Attitude		−0.098*** (0.023)
Attitude * Internet duration		0.058* (0.036)
Attitude* Internet duration square		0.013*** (0.004)
Control variables	Yes	Yes
*R*-squared	0.14	0.12

***p < 0.01, **p < 0.05, *p < 0.1.

## 6 Discussion

Based on the 2018 and 2020 CFPS surveys, this study has examined the casual relationship between Internet use and mental health by employing the PSM-DID method in a Chinese setting. Using the PSM-DID model, we found that, after implementing a control model to take into account demographic variables, Internet use had a significant effect on alleviating the levels of depression in middle-aged and older adults. This is in line with previous research (Quintana et al., 2018; Du et al., 2023). Although the COVID-19 pandemic had negative impact upon the health of middle-aged and older adults, levels of depression among Internet users was significantly lower than that of non-Internet users. One possible reason for the divergent levels of depression in the two groups during the COVID-19 pandemic is that the outbreak of pandemic generated shock in older people, which may have had an initial impact on mental health. However, as the first few months of the pandemic progressed, the Internet brought to light more information about the disease and methods of preventing and treating it. Consequently, older adults displayed the lowest prevalence of psychological distress (Beauchamp et al., 2021; López et al., 2020), although this demographic was identified as being particularly at risk of isolation and diminished wellbeing (Banerjee, 2020). As such, they represented the focus of intervention in this trial.

Online entertainment has a significant impact on depression level. Numerous researches have shown that engaging in leisure activities promotes mental health (Takiguchi et al., 2023; Santini et al., 2020; Fancourt et al., 2021). Our empirical results consist with previous studies. Social media use in middle-aged and older adults can improve their mental health. Our findings reinforce previous findings, providing new evidence that social media usage can reduce the impression level during the COVID-19 pandemic. This suggests that social networking can be used as a potential therapy to alleviate depression level. For middle-aged and older adults, when they were geographically separated with their family members and friends during the COVID-19 pandemic, online communication were much more important channels to substitute the face-to-face contact. As a result, social networking can increase chances of social exchange and reduce social isolation and loneliness. Online shopping and online learning have no significant effect on depression level. The conclusion contradicts with previous literature (McKinlay et al., 2021). The possible explanation is that the proportion of online learning (2.84% of sample in 2018; 1.6% of sample in 2020) and online shopping (3.05% of sample in 2018; 3.86% of sample in 2020) among middle-aged and older adults are smaller than that of other age groups, respectively.

In addition, the impact of Internet use on the mental health varied between rural and urban residents. Due to the inadequate IT infrastructure and socioeconomic support resources in rural regions, the urban and rural residents may not equally enjoy the benefit of Internet use (Hong and Cho, 2017; Wang et al., 2019; Sun et al., 2022). Our study shows that digital divide in a country with a dual economy like China is foremost a question of differences in the chance of access to the Internet in rural and urban areas. The disparity in Internet use between urban and rural residents results in substantial differences in their levels of mental health. Rural residents have limited access to social opportunities, healthcare resources due to underdeveloped economy and healthcare system. The disparities could lead to variations in the impact of Internet use on depression between rural and urban older adults (Liao et al., 2020; Liu and Griffiths, 2011). Therefore, greater marginal gains could be realized by enhancing the digital capacity and narrowing the digital divide among rural residents. However, the divide is predominantly technological, yet the challenges in bridging it appear to stem from a complex combination of political and financial obstacles. Policy recommendation is to improve the digital infrastructure in rural areas and provide more opportunities for rural residents to access the Internet.

Furthermore, this research used perception of Internet as a mediating variable to explore its mediating effect on the relationship between time spent online and depression in middle-aged and older adults. Using the Internet can enable middle-aged and older adults to obtain knowledge about health and health services, and alleviate depression. On the flipside, excessive Internet use will negatively impact mental health. Excessive use of the Internet can also serve as an indicator of likely outcomes in psychosocial health in areas including as loneliness, phlegmaticism and impulsive behavior. Evidence confirms the U curve-shaped relationship between time spent online and mental health. Based on cognitive emotion theory and emotion-action link, this study has explored the mechanism behind the relationship between time spent online and mental health by using moderator effects model in middle-aged and older adults. The results showed that the perception of Internet enhances the impact of time spent online. It tends to increase the positive effects of Internet usage on reducing depression levels for online activities or information and content obtained from the Internet enrich life experiences and expand the scope of social interaction among middle-aged and older adults. This serves in turn to improve their psychological wellbeing (Nie et al., 2017). Nevertheless, beyond a certain threshold, this attitude can shift to amplify the negative influence of time spent online upon the symptoms of depression. The greater the importance individuals attach to the Internet, the more time they spend on it and the higher the incidence of Internet addiction or problematic usage. The results align with the contention that the Internet exerts an indirect influence on depressive symptoms (Wu B. et al., 2023).

Promoting digital literacy and Internet usage among the middle-aged and senior population brings with it a plethora of benefits. Particularly, cultivating digital health literacy is of paramount importance. Individuals possessing a higher degree of digital health literacy exhibit reduced levels of anxiety and depression (Xu et al., 2022), and they are more likely to experience optimal shared decision-making and improved capability wellbeing (Xu et al., 2021). This is attributed to their ability to harness an abundance of health resources available online, thereby facilitating superior self-management. Notably, amidst pandemics, such as the one induced by COVID-19, this proficiency aids in mitigating adverse emotional states like depression and insomnia, while also enabling the proficient identification and utilization of online healthcare resources to uphold and bolster mental wellbeing (Yang et al., 2021). This initiative nurtures a deeper acceptance and active participation in digital healthcare solutions, a sector that has witnessed exponential growth and global proliferation in response to the COVID-19 pandemic. The results of this study suggest that investments in digital capacity for middle-aged and older adults, including fostering positive perceptions of the Internet (e.g., increasing trust) and advancing digital literacy (e.g., preventing problematic Internet use and cultivating digital health literacy) can provide substantial benefits in terms of preserving mental health, lowering depression scores and promoting psychological wellbeing. All of this proves particularly valuable in ordinary times and more so during times of crisis.

Admittedly, there are some limitations to this study. First of all, the mental health of middle-aged and older adults is affected by variable factors. Although the investigators implemented controlled variables such as age, gender, educational levels, and self-assessed health, it was still difficult to exclude the possible effects of other factors, including macrolevel social factors. Secondly, only two waves of CFPS data were used in the PSM-DID model. If more waves of data from pre- and post- the COVID-19 pandemic were included, the impact of Internet use on the mental health of middle-aged and older adults would be as pronounced as expected. Thirdly, the measurement of depression in CFPS is in the form of self-report, and the scoring may not be objective. Fourthly, due to the constraints imposed by limited data of CFPS, only four types of Internet use were analyzed, including online entertainment, online learning, online shopping and social networking, if more types of Internet use such as online health-related information seeking, telemedicine are analyzed, impact of Internet use on mental health would be more persuasive. Lastly, in terms of the mechanism of time spent online and health, only attitudes toward the Internet attitude as a mediator was tested. It may very well be that there are additional channels which influence the U curve-shaped relationship.

## Data Availability

Publicly available datasets were analyzed in this study. This data can be found at: http://www.isss.pku.edu.cn/english/index.htm?CSRFT=SP95-ZJ68-4JJL-G0D2-9U75-DZ9F-K0JI-L6BG.
